# Plant‐Beneficial *Streptomyces thermocarboxydus* S3 Mitigates Heat Stress in Hydroponically Grown Lettuce

**DOI:** 10.1155/sci5/3095586

**Published:** 2026-02-23

**Authors:** Benyapa Kitwetch, Yupa Chromkaew, Wasu Pathom-aree

**Affiliations:** ^1^ Multidisciplinary and Interdisciplinary School, Chiang Mai University, Chiang Mai, 50200, Thailand, cmu.ac.th; ^2^ Department of Plant and Soil Sciences, Faculty of Agriculture, Chiang Mai University, Chiang Mai, 50200, Thailand, cmu.ac.th; ^3^ Department of Biology, Faculty of Science, Chiang Mai University, Chiang Mai, 50200, Thailand, cmu.ac.th; ^4^ Department of Biology, Faculty of Science, Center of Excellence in Microbial Diversity and Sustainable Utilization, Chiang Mai University, Chiang Mai, 50200, Thailand, cmu.ac.th

**Keywords:** climate resilient agriculture, hydroponic lettuce, heat stress tolerance, oxidative stress mitigation, plant growth-promoting actinobacteria, *Streptomyces thermocarboxydus*

## Abstract

Climate change presents a significant threat to global agriculture by increasing abiotic stresses that negatively impact crop yields. Lettuce (*Lactuca sativa*), a cool‐season crop, is particularly vulnerable to heat stress, which accelerates metabolism and increases respiration rates beyond photosynthetic capacity, ultimately leading to growth and yield reduction. In hydroponic systems, elevated temperatures further impair plant development by altering nutrient solubility and availability, resulting in deficiencies. Microbial bioinoculants offer a sustainable and ecofriendly strategy to mitigate heat stress and enhance plant performance in crop production. Actinobacteria, in particular, are recognized for their plant growth‐promoting properties. This study evaluates the effectiveness of *Streptomyces thermocarboxydus* S3 in enhancing hydroponic lettuce growth under heat stress conditions. Inoculation with *S. thermocarboxydus* S3 significantly improved key growth parameters, including fresh weight, dry weight, number of leaves, and chlorophyll content. The strain also induced the accumulation of osmoprotective compounds, such as proline and total soluble sugar (TSS), contributing to cellular protection under thermal stress Additionally, *S. thermocarboxydus* S3 reduced hydrogen peroxide (H_2_O_2_) levels, indicating a potential role in oxidative stress mitigation and activation of plant defense responses. Root colonization assays confirmed the strain’s ability to establish itself in the lettuce roots, supporting its applicability for long‐term application. These findings highlight *S. thermocarboxydus* S3 as a promising bioinoculant for promoting hydroponic lettuce growth under heat stress, offering a sustainable approach to crop production in the context of changing climate.

## 1. Introduction

Climate change has emerged as one of the most critical global challenges, causing unstable weather patterns, extreme temperatures, and disruptions to natural ecosystems. According to the National Oceanic and Atmospheric Administration (NOAA), the last decade has been the warmest on record, highlighting the urgent need to address the consequences of rising global temperatures. These climatic shifts pose serious threats to agricultural productivity and global food security by disrupting key physiological processes in plants [1], particularly photosynthesis, ultimately reducing crop yields [2]. Additionally, climate change intensifies the scarcity of surface water, accelerates the depletion of aquifers, and increases the frequency and intensity of abiotic stresses such as drought, salinity, flooding, and heat stress [3]. These environmental stressors also give rise to secondary challenges including pest outbreaks [4] and soil degradation [5], collectively threatening the sustainability of global food systems.

High temperatures can severely impair plant growth and development, sometimes resulting in irreversible damage or plant death [6]. To survive under such conditions, plants have evolved a range of adaptive mechanisms that regulate key physiological processes, including photosynthesis, respiration, and water metabolism [7]. However, heat stress often reduces photosynthetic efficiency, shortens the plant life cycle, and lowers productivity. One of the major physiological consequences of heat stress is membrane instability [8]. Extreme heat increases the kinetic energy of biomolecules, weakening chemical bonds and disrupting membrane lipids, thereby compromising membrane function leading to instability [9, 10]. Additionally, heat stress triggers the overproduction of reactive oxygen species (ROS), such as singlet oxygen, superoxide radicals, hydrogen peroxide (H_2_O_2_), and hydroxyl radicals, which cause oxidative damage to cellular structures [11]. At the morphological level, heat stress leads to reductions in growth duration, leaf area, seed germination, and grain quality, all of which negatively affect overall crop performance [12].

Lettuce (*Lactuca sativa* L.), a cool‐season crop, is highly sensitive to elevated temperatures, with optimal growth occurring between 15°C and 25°C [13]. Temperatures exceeding this range adversely affect lettuce development, resulting in reduced leaf expansion, premature bolting, and declines in both yield and quality [14, 15]. In hydroponic systems, high ambient temperatures can further impair nutrient uptake, disrupt photosynthetic efficiency, and induce oxidative damage, all of which compromise plant performance [16]. Additionally, heat stress induces both oxidative damage and osmotic damage in plants. It promotes the accumulation of ROS, particularly superoxide anions, which are subsequently converted to H_2_O_2_. This build‐up causes cellular damage, affecting membranes, proteins, and nucleic acids. H_2_O_2_ serves not only as a marker of oxidative stress but also as a signaling molecule involved in regulating transpiration and water‐use efficiency [17]. These physiological limitations highlight the urgent need for innovative approaches to improve heat tolerance in lettuce, particularly in hydroponic production systems.

Plant growth‐promoting bacteria (PGPBs), including members of the phylum Actinomycetota, such as *Streptomyces*, have emerged as promising bacteria to enhance plant resilience to abiotic stresses. These beneficial microbes improve plant growth under stress conditions through multiple mechanisms, including the production of phytohormones, promotion of nutrient uptake, and modulation of stress‐responsive signaling pathways [18–21]. Certain *Streptomyces* strains have demonstrated the ability to promote plant growth and induce resistance against various abiotic stresses such as drought [22], salinity [23], nutrient limitation [19], and heat [24].

Thermotolerant PGPBs mitigate heat stress in plants through multiple mechanisms, such as the upregulation of heat shock protein genes, accumulation of osmolytes, modulation of antioxidant enzyme activity, and regulation of hormonal responses [25]. Phytohormones, such as jasmonic acid (JA), ethylene, indole‐3‐acetic acid (IAA), gibberellic acid (GA), salicylic acid (SA), abscisic acid (ABA), and melatonin play critical roles in regulating plant responses to heat stress by promoting seed germination, seedling growth, and stress tolerance [26]. In particular, IAA has been shown to enhance plant tolerance to heat stress by reducing oxidative damage caused by ROS [27].

Despite growing evidence supporting the potential of PGPBs, their application in hydroponic systems, particularly under high‐temperature stress, remains limited. Recent study has shown that inoculation with thermotolerant PGPBs can improve lettuce growth and nutrient uptake in hydroponic systems exposed to heat stress, potentially by altering stomatal movement and chlorophyll fluorescence parameters [28]. However, the specific roles and mechanisms of Streptomyces spp. in mitigating heat stress in hydroponically grown lettuce are still not fully understood. This study aims to evaluate the potential of plant‐beneficial actinobacterium, *S. thermocarboxydus* S3, to mitigate the adverse effects of heat stress on hydroponically grown lettuce. In addition, the mechanisms by which actinobacteria confer thermotolerance are also investigated with the ultimate goal of enhancing crop resilience and supporting the development of sustainable hydroponic agriculture under climate change conditions.

## 2. Materials and Methods

### 2.1. Actinobacteria, Plant Material, and Nutrient Solution

*S. thermocarboxydus* strain S3, previously isolated from spores of the arbuscular mycorrhiza, *Funneliformis mosseae*, was used in this study [29]. The strain was routinely cultured on International Streptomyces Project 2 (ISP2) agar and incubated at 28 ± 2°C. Lettuce (*Lactuca sativa* L. var. *longifolia*) seeds were obtained from the Vegetable Seed Production and Organic Farming Learning Center, Maejo University, Thailand. The hydroponic nutrient solution was obtained from Kitsuwan Farm, Nonthaburi, Thailand, consisted of solution A (57.5 g/L of Ca(NO_3_)_2_, 1 g/L of 7% Fe‐DTPA, and 2 g/L of 13.2% Fe‐EDTA) and solution B (30 g/L of KNO_3_, 25 g/L of MgSO_4_, 13.25 g/L of KH_2_PO_4_, 2.5 g/L of Dissolvine ABC EDTA, and 0.5 g/L of 13% Mn‐EDTA). Both solutions were prepared and combined according to the manufacturer’s instructions.

### 2.2. Inoculant Preparation

Lettuce seeds were sown in the growing tray (4 cm × 4 cm × 5.25 cm) containing a growing substrate (perlite and vermiculite in a 3:1 ratio) and cultivated under greenhouse conditions during summer season from March 2024–May 2024 at the Faculty of Agriculture, Chiang Mai University, Thailand. The experiment consisted of two treatments: (1) an uninoculated control and (2) inoculation with *S. thermocarboxydus* S3. Seedlings were watered daily for 7 days before transplanted into the nutrient film technique (NFT) hydroponic system. The electrical conductivity (EC) of the nutrient solution was maintained at 1800 μS/cm throughout the experiment. After 45 days of cultivation, lettuce plants were harvested and the following growth parameters were measured: number of leaves, shoot and root length (cm), and fresh weight and dry weight (*g*). Dry weight was determined after oven‐drying the samples at 70°C until a constant weight was achieved.

### 2.3. Experimental Design

*S. thermocarboxydus* S3 was grown on Seino agar containing 10 g/L of soluble starch, 3 g/L of casein enzymatic hydrolysate, 1 g/L of yeast extract, 1 g/L of meat extract, 3 g/L of CaCO_3_, and 18 g/L of agar at 28 ± 2°C for 7 days to promote spore formation. A spore suspension was prepared by scraping fully formed spores from the agar surface and suspended in sterile distilled water. The spore suspension was adjusted to obtain an optical density (OD_600_) of 1.00, which is equivalent to 10^8^ spore/mL. This spore suspension was used for seed inoculation in the subsequent greenhouse experiment. Surface sterilization of lettuce seeds was carried out using the method described by Kitwetch et al. [19]. Briefly, seeds were soaked in 70% (v/v) ethanol for 1 min, followed by 1.2% (v/v) sodium hypochlorite (NaClO) solution for 12 min, and rinsed 3 times with sterile distilled water for 1 min. The effectiveness of the sterilization method was validated by placing surface sterile seeds on nutrient agar (containing 5 g/L of peptone, 5 g/L of NaCl, 2 g/L of yeast extract, 1 g/L of meat extract, and 18 g/L of agar) and incubated at room temperature for 48 h. The surface sterile lettuce seeds were incubated in spore suspension of *S. thermocarboxydus* S3 (10^8^ spores/mL) for 3 h shaking at 120 rpm before sowing. Seeds treated with sterile distilled water served as uninoculated control.

### 2.4. Biochemical Analysis

#### 2.4.1. Chlorophyll and Carotenoid Content

Chlorophyll and carotenoid contents were determined to follow the standard method of Arnon [30]. One gram of fresh lettuce leaves was cut into small pieces and soaked in 10 mL of 80% (v/v) acetone for 48 h in the dark. The supernatant was collected and measured at absorbances of 480, 645, and 663 nm. The chlorophyll and carotenoid contents were calculated using the following equation:

(1) Total chlorophyllmgg fresh weight=20.2802×OD645+.×OD663×V1000×W,

(2) Carotenoidsmgg fresh weight=4.6950.268×OD480−×V1000×W,

where V is the volume of the extract (mL) and W is the fresh weight of the tissue sample (*g*).

#### 2.4.2. H_2_O_2_ Content

H_2_O_2_ content was performed following Kitwetch et al. [19]. Briefly, 500 mg of fresh lettuce leave was homogenized in 3 mL of 0.1% (*w*/*v*) trichloroacetic acid on ice bath. The homogenate was centrifuged at 12,000 rpm for 10 min at 4°C. Then, 500 μL of the supernatant was mixed with 500 μL of 10 mM potassium phosphate buffer (pH 7.0) and 1000 μL of 1 M KI solution. The reaction mixture was spectrophotometrically measured at an absorbance of 390 nm. H_2_O_2_ content was estimated from a standard curve prepared with known concentrations of H_2_O_2_ and expressed as mg/g fresh weight.

#### 2.4.3. Total Soluble Sugar (TTS) Content

TSS content was measured following Rangseekaew et al. [18]. A 250 mg of fresh lettuce leaves was homogenized in 3 mL of 80% (*v*/*v*) ethanol and incubated at 75°C for 15 min. The homogenate was centrifuged at 12,000 rpm for 10 min. Then, 250 μL of the supernatant was mixed with 250 μL of 80% ethanol, followed by 2.5 mL of concentrated H_2_SO_4_ and 500 μL of 5% (*v*/*v*) phenol. The reaction mixture was incubated at room temperature for 20 min and an absorbance measured at of 490 nm. The TSS content was calculated from a standard curve prepared with known concentration of glucose and expressed as mg/g fresh weight.

#### 2.4.4. Proline Content

Proline content was determined according to Rangseekaew et al. [18]. A 250 mg of fresh lettuce leaves was homogenized in 3 mL of 80% (*v*/*v*) ethanol and centrifuged at 12,000 rpm for 10 min. The reaction mixture containing 200 μL of supernatant, 300 μL of distilled water, and 2 mL of ninhydrin solution was incubated in a boiling water bath for 1 h. The reaction was stopped in an ice bath and 6 mL of toluene was added followed by vortexing for 20 s. The top organic layer was collected and absorbance was measured at 520 nm. Proline content was calculated from a standard curve prepared with known concentration of L‐proline and expressed as mg/g fresh weight.

#### 2.4.5. Nonenzymatic Antioxidant Extraction and Assays

Nonenzymatic antioxidants were determined following the method of Liu et al. [31]. Briefly, 0.1 g of lyophilized lettuce tissue was extracted with 10 mL of acetone: water (70: 30, *v*/*v*) for 24 h. The extract was centrifuged at 12,000 rpm for 10 min, and the supernatant was stored in the dark at 4°C for subsequent analyses.

#### 2.4.6. DPPH Radical Scavenging Activity

The 2,2‐diphenyl‐1‐picrylhydrazyl (DPPH) assay was carried out following Miliauskas et al. [32]. A 100 μL of lettuce leave extracts was mixed with 0.5 μL of 0.5 mg/mL DPPH solution and incubated in the dark for 20 min. The absorbance of the reaction mixture was measured at 517 nm. DPPH radical scavenging activity was expressed as mg gallic acid/g lettuce dry weight (mg GAE/g DW).

#### 2.4.7. ABTS Radical Scavenging Activity

The 2,2′‐azino‐bis(3‐ethylbenzothiazoline‐6‐sulfonic acid) diammonium salt (ABTS) radical cation decolorization assay was carried out [32]. ABTS stock solution was prepared by mixing 7.4 mM ABTS solution with 2.45 mM potassium persulfate (1:1, *v*/*v*) and incubated in the dark at room temperature for 12 h. For the assay, 5 μL of lettuce leave extract was mixed with 195 μL of ABTS solution and incubated for 10 min in the dark. The absorbance of the reaction mixture was measured at 734 nm and results were expressed as mg Trolox equivalent per *g* of dry weight (mg TE/g DW).

#### 2.4.8. Total Phenolic Content

Total phenolic content was determined using the Folin–Ciocalteu method [32]. A 20 μL aliquot of the extracts was mixed with 100 μL of 10% Folin–Ciocalteu solution and incubated at room temperature for 8 min. Then, 80 μL of 7.5% Na_2_CO_3_ solution was added and incubated in the dark at room temperature for 30 min. The absorbance of the reaction mixture was measured at 765 nm. Total phenolic content was expressed as mg gallic acid/g lettuce dry weight.

#### 2.4.9. Nutrient Analysis

Lettuce samples were dried in a hot air oven at 70°C for 48 h. The dried lettuce was finely ground using a multifunctional grinder and filtered through 0.5 mm filter.

#### 2.4.10. Nitrate Content

Nitrate content was determined following the method of [33]. Briefly, 0.1 g of lettuce powder was suspended in 10 mL of deionized water and incubated in a water bath at 80°C for 30 min to extract nitrate. The extract was centrifuged at 13,000 rpm for 10 min. Then, 200 μL of the supernatant was mixed with 19 mL of 8% NaOH and 0.8 mL of 5% (*w*/*v*) SA. The absorbance of the reaction mixture was measured at 410 nm. Nitrate content was calculated using a standard curve prepared with known concentration of potassium nitrate (KNO_3_) and expressed as g/kg dry weight.

#### 2.4.11. Ammonium Content

For ammonium concentration, 0.5 g of lettuce powder was digested with 25 mL of concentrate H_2_SO_4_ in block‐digestion unit (Gerhardt‐VAP 450, Germany) at 220°C for 1 h. The digested sample was filtered through Whatman No. 5 and the filtrate was kept for analysis. A 0.2 mL aliquot of the filtrate was mixed with 3 mL of solution I (a mixture of 4% EDTA, 3.2 M SA, and 0.05% sodium nitroprusside in a 1:10:100 ratio) and 5 mL of solution II (a mixture of 1 M sodium hypochlorite and Na_2_HPO_4_ buffer (pH 12.3) in a 1:4 ratio). The mixture was vortexed for 5 s and incubated at room temperature for 2 h. Absorbance was measured at 660 nm. Ammonium content was calculated from a standard curve prepared with known concentration of ammonium sulfate ((NH_4_)_2_SO_4_) and expressed as g/kg dry weight.

#### 2.4.12. Phosphorus (P), Potassium (K), Calcium (Ca), and Magnesium (Mg) Content

For the determination of P, K, Ca, and Mg, 0.5 g of ground lettuce powder was digested with 25 mL of a nitric–perchloric acid mixture (HNO_3_:HClO_4_, 6:1, v/v) in the block digestion unit (Gerhardt‐VAP 450, Germany) at 220°C for 1 h. The digested sample was filtered through Whatman No. 5 filter paper, and the filtrate was stored in clean bottles for analysis.

P content was determined using Bray II method [34]. The filtrate (1 mL) was mixed with 1 mL of reagent mixture containing 0.01 M NH_4_VO_3_ and 0.02 M (NH_4_)_6_Mo_7_O_24_ and incubated for 20 min. The absorbance of the reaction mixture was measured at 470 nm. P concentration was calculated using a KH_2_PO_4_ standard curve and expressed as g/kg dry weight. K, Ca, and Mg contents were analyzed using atomic absorption spectrometer (Contr800, Analytik Jena, Germany) and reported as g/kg dry weight.

#### 2.4.13. Root Colonization

At the end of experiment (Day 45), lettuce root from the hydroponic system was surface sterilized with 1% NaHClO for 1 min, followed by 70% ethanol for 1 min, and washed with sterile distilled water for 1 min twice. 10 g of sterile lettuce root was grounded in 90 mL of 0.85% NaCl solution and serially diluted 10‐fold from 10^−1^ to 10^−6^. Aliquots of each dilution were spread on Seino agar plates supplemented with 100 μg/mL nalidixic acid and 100 μg/mL ketoconazole and incubated at 28 ± 2°C for 14 days. Presumptive colonies of *S. thermocarboxydus* S3 were counted, expressed as CFU/g root fresh weight, and confirmed by 16S rRNA gene sequencing. Dried‐root sample was coated with gold and the colonization of *S. thermocarboxydus* S3 was observed under scanning electron microscope (SEM) (JSM‐5910 LV scanning electron microscope, JEOL Ltd., Japan).

#### 2.4.14. Statistical Analysis

All experiments were performed in triplicate, and the results are expressed as mean ± standard deviation. Statistical significance was assessed using the *Student′s t-test*, with differences considered significant at *p* < 0.05. Additionally, linear regression analysis was used to evaluate the relationship between H_2_O_2_ levels and heat stress response indicators, including proline, TSS, and DPPH radical scavenging activity. Pearson’s correlation coefficient was calculated to determine the strength and direction of associations among various heat stress parameters. Statistical analyses were performed using IBM SPSS Statistics version 22.0 (IBM Corp., Armonk, NY, USA), and correlation analyses were conducted using *R* software (version 4.4.3).

## 3. Results

### 3.1. Morphological Parameters

Lettuce was cultivated in a NFT hydroponic system using a full‐strength nutrient solution under outdoor greenhouse conditions from 16 April to 31 May 2023. During the experimental period, temperatures ranged from a minimum of 23.3°C to a maximum of 54°C, with an average temperature of 38.3°C. The experiment was conducted under natural daylight without artificial lighting and the average relative humidity was recorded at 60.18%. Morphological and biochemical parameters were investigated after 45 days of cultivation. Lettuce plants inoculated with *S. thermocarboxydus* S3 showed superior overall appearance than uninoculated controls, indicating a positive effect of microbial inoculation under heat stress conditions (Figure 1).

FIGURE 1Representative appearance of 45 day‐old hydroponically grown lettuce subjected to heat stress conditions. (a) Uninoculated control plants and (b) plants inoculated with *S. thermocarboxydus* S3.

**Figure:**
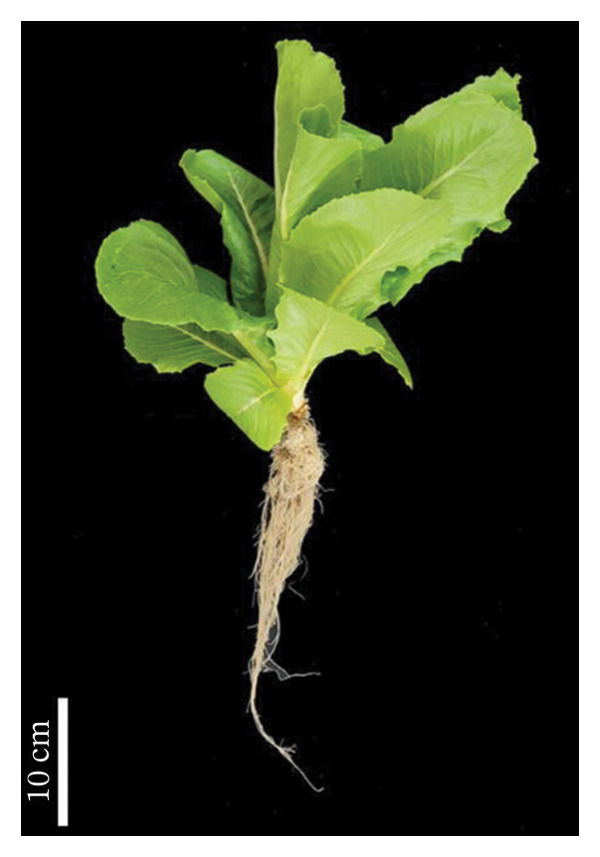
(a)

**Figure:**
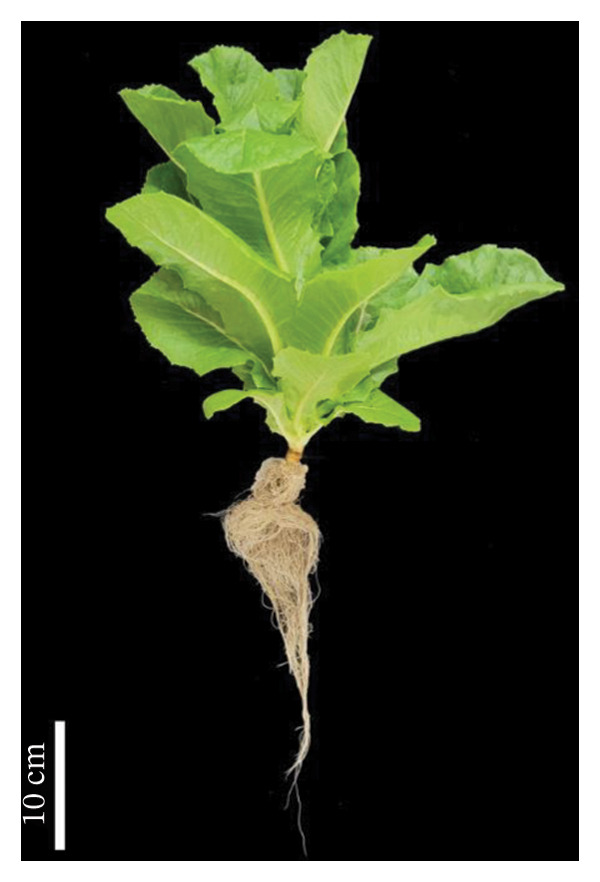
(b)

Lettuce plants inoculated with *Streptomyces thermocarboxydus* S3 exhibited a 12% increase in fresh weight and an 18% increase in dry weight compared to uninoculated controls (Figure 2(a)). However, these differences were not statistically significant. Shoot length in the inoculated lettuce was 6% lower than in the control group, whereas root length was 16% higher, though neither change reached statistical significance (Figure 2(b)). Notably, the number of leaves in inoculated lettuce was significantly higher by 20% than in the uninoculated group (*p* < 0.05) (Figure 2(c)), suggesting a potential enhancement in vegetative growth induced by the inoculation of *S. thermocarboxydus* S3 under heat stress conditions.

FIGURE 2Effects of *S. thermocarboxydus* S3 inoculation on morphological parameters of 45 day‐old hydroponically grown lettuce under heat stress conditions. (a) Fresh and dry weight; (b) shoot and root length; (c) number of leaves. Data represent the mean values of three replicates. Different letters indicate significant differences between treatments according to *Student′s t*‐test (*p* < 0.05).

**Figure:**
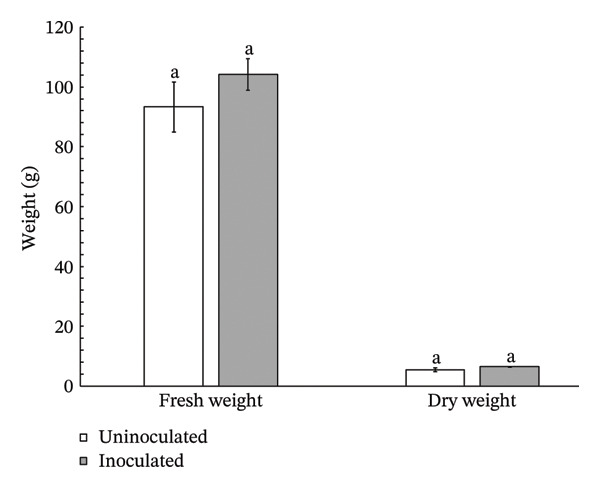
(a)

**Figure:**
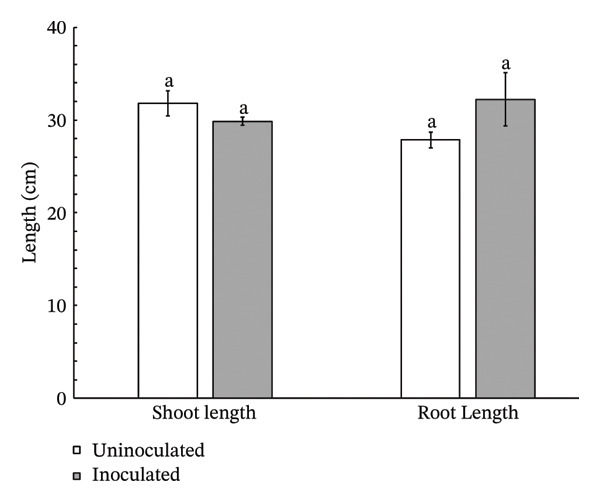
(b)

**Figure:**
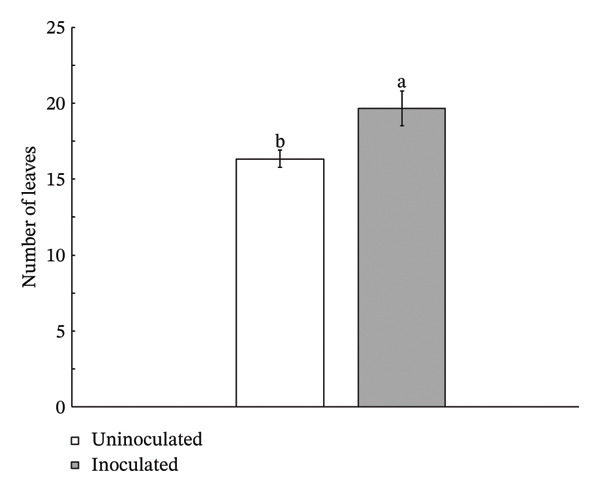
(c)

### 3.2. Biochemical Parameters

Inoculation with *S. thermocarboxydus* S3 positively influenced several biochemical parameters in lettuce under heat stress conditions. Total chlorophyll and carotenoid contents in inoculated lettuce were 11% and 7% higher, respectively, compared to uninoculated controls (Figure 3(a)). Moreover, the accumulation of osmoprotectants such as proline and TSS increased significantly by 24% and 37%, respectively, in inoculated lettuce (Figure 3(b)), indicating improved osmotic adjustment. The concentration of H_2_O_2_, a key ROS in lettuce, was decreased by 47% as compared with uninoculated lettuce (Figure 3(c)). Antioxidant activity, as measured by DPPH radical scavenging, which primarily reflects lipophilic antioxidants, decreased by 11% in inoculated lettuce (Figure 3(d)). In contrast, ABTS scavenging activity, which reflects both hydrophilic and lipophilic antioxidant capacity, increased by 5% (Figure 3(d)) and total phenolic content remained unchanged between treatments. These results suggest that *S. thermocarboxydus* S3 promotes biochemical adaptations that enhance heat stress tolerance, particularly through osmolyte accumulation and regulation of oxidative stress.

FIGURE 3Effects of *S. thermocarboxydus* S3 inoculation on biochemical parameters of 45 day‐old lettuce. (a) Total chlorophyll and carotenoid contents; (b) proline and total soluble sugar contents; (c) hydrogen peroxide content; (d) nonenzymatic antioxidants including DPPH, ABTS, and total phenolic compounds. Data represent the mean values of three replicates. Different letters indicate significant differences between treatments according to *Student′s t*‐test (*p* < 0.05).

**Figure:**
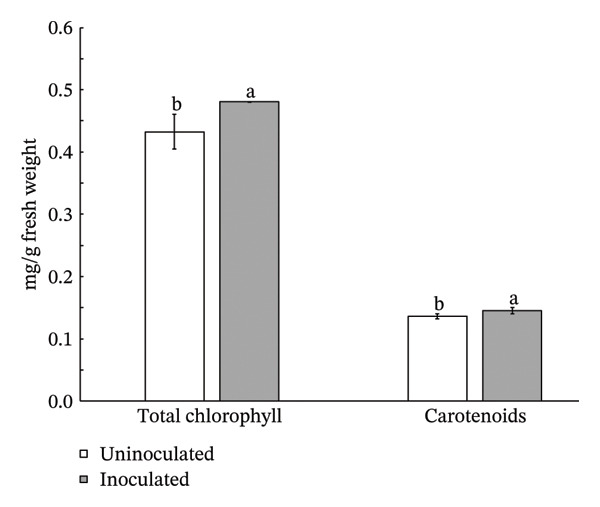
(a)

**Figure:**
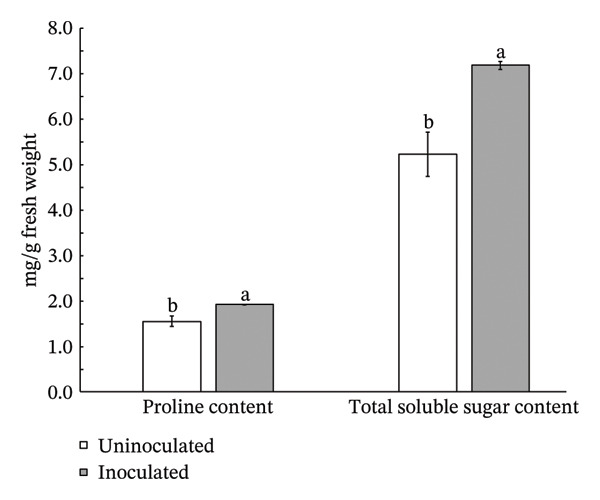
(b)

**Figure:**
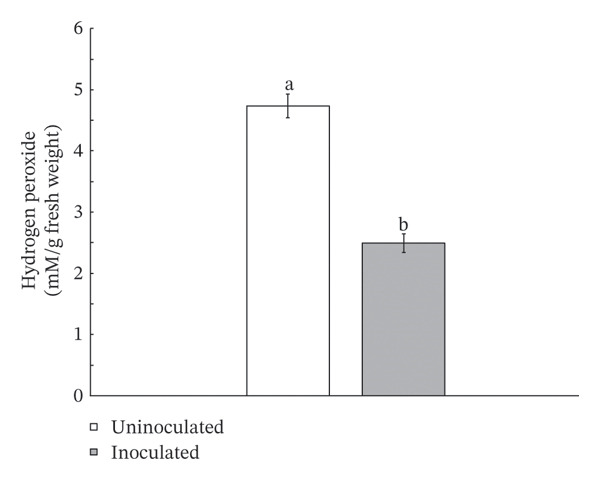
(c)

**Figure:**
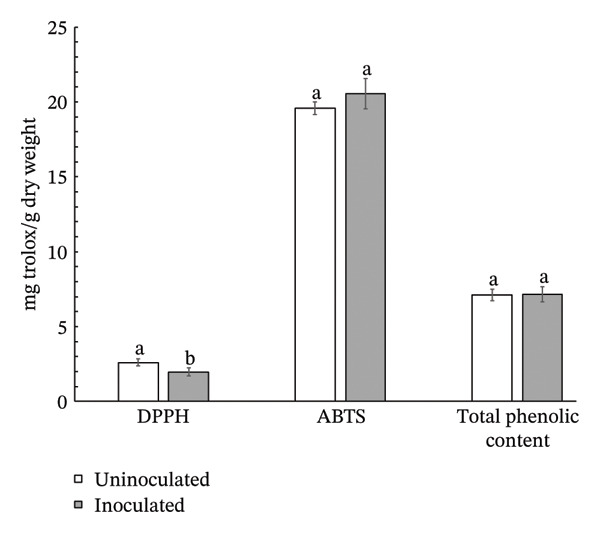
(d)

### 3.3. Nutritional Parameters

The nutritional quality of 45 day‐old hydroponic lettuce under heat stress did not differ significantly between uninoculated and *S. thermocarboxydus* S3‐inoculated treatments (Table 1). A trend toward higher nitrate (NO_3_^−^) accumulation and lower ammonium (NH_4_^+^) content was observed in inoculated lettuce compared to uninoculated control. Similarly, P accumulation in inoculated lettuce was slightly higher, whereas K accumulation was marginally lower. Regarding micronutrients, higher Ca accumulation was observed in inoculated lettuce but lower in Mg level relative to the uninoculated control. Although these variations were not statistically significant, they suggest subtle nutrient modulation in response to microbial inoculation. These findings suggest that the use of *S. thermocarboxydus* S3 does not adversely affect the nutritional composition of hydroponically grown lettuce under heat stress, supporting its potential as a bioinoculant without compromising crop quality.

**TABLE 1:** Nutrient contents of 45 day‐old hydroponic lettuce under heat stress condition.

Treatment	NO_3_^-^	NH_4_^+^	P	K	Ca	Mg
	g/kg DW	g/kg DW	Mg/kg DW	g/kg DW	g/kg DW	g/kg DW
Uninoculated	87.41 ± 16.14^a^	37.02 ± 2.94^a^	550.04 ± 7.46^a^	64.11 ± 3.57^a^	30.03 ± 1.08^a^	4.46 ± 0.20^a^
Inoculated	102.22 ± 3.71^a^	37.50 ± 1.67^a^	549.67 ± 22.22^a^	59.93 ± 2.29^a^	30.39 ± 0.57^a^	4.31 ± 0.36^a^

*Note:* Data represent mean values of three replicates ± SD. The different superscript letters within the same column indicate significant differences between treatments according to *Student′s t-test* (*p* < 0.05).

^a^Refers to no significant difference between treatments.

### 3.4. Correlation Between H_2_O_2_ and Heat Stress Responses

**FIGURE 4:**
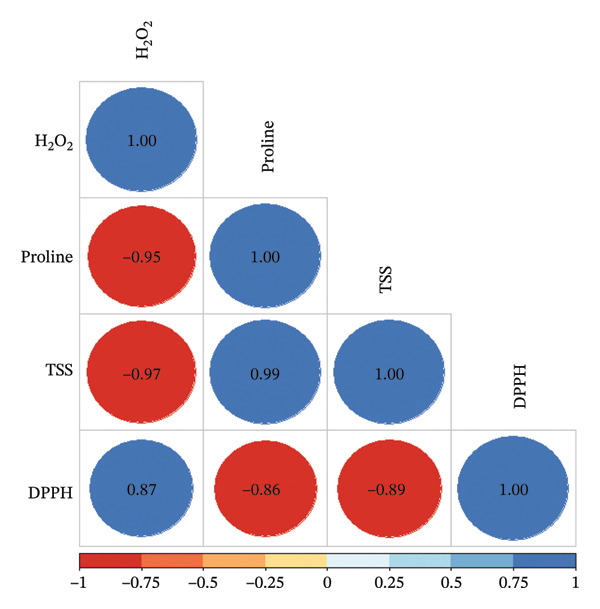
Pearson’s correlation matrix showing the relationships among hydrogen peroxide (H_2_O_2_) content, proline accumulation, total soluble sugars (TSSs), and DPPH radical scavenging activity in hydroponically grown lettuce under heat stress conditions. Blue circles indicate positive correlations, while red circles indicate negative correlations. The size and color intensity of each circle represents the strength of the correlation, and the numerical values inside the circles correspond to Pearson’s correlation coefficients (*r*).

Pearson’s correlation analysis was conducted to examine the relationships among key biochemical responses under heat stress conditions (Figure 4). H_2_O_2_ levels exhibited strong negative correlations with compatible solutes, in particular proline (*r* = −0.949, *p* < 0.01) and TSS (*r* = −0.967, *p* < 0.01), indicating that elevated osmolyte accumulation is associated with reduced oxidative stress. Conversely, H_2_O_2_ showed a significant positive correlation with DPPH radical scavenging activity (*r* = 0.867, *p* < 0.05), suggesting a potential compensatory antioxidant response to elevated ROS levels. Proline content showed a highly significant positive correlation with TSS (*r* = 0.992, *p* < 0.001), supporting their coordinated role in osmotic adjustment. However, proline was negatively correlated with DPPH activity (*r* = −0.856, *p* < 0.05), as was TSS (*r* = −0.890, *p* < 0.05).

### 3.5. Root Colonization of *S. thermocarboxydus* S3 in Hydroponic Lettuce Root

A total of 3.2 × 10^4^ CFU/mL of *S. thermocarboxydus* S3 was reisolated from the roots of inoculated lettuce, confirming successful colonization under hydroponic conditions. The identity of the reisolated strain was confirmed by 16S rRNA gene sequencing, which showed 99.93% similarity to the type strain *S. thermocarboxydus* DSM 44293^T^. SEM provided further evidence of colonization. SEM images revealed filamentous structures characteristic of Streptomyces on the root surfaces of inoculated plants (Figure 5(b)). In contrast, no microbial structures were observed on the roots of uninoculated control plants, indicating the absence of natural microbial colonization under the experimental conditions (Figure 5(a)). These findings confirm the ability of *S. thermocarboxydus* S3 to effectively colonize lettuce roots in hydroponic systems, even under heat stress, supporting its potential as a bioinoculant.

FIGURE 5Scanning electron micrographs of hydroponics lettuce roots after 45 days. (a) Uninoculated lettuce; (b) *S. thermocarboxydus* S3‐inoculated lettuce. Red arrows indicate filamentous cells of *S. thermocarboxydus* S3.

**Figure:**
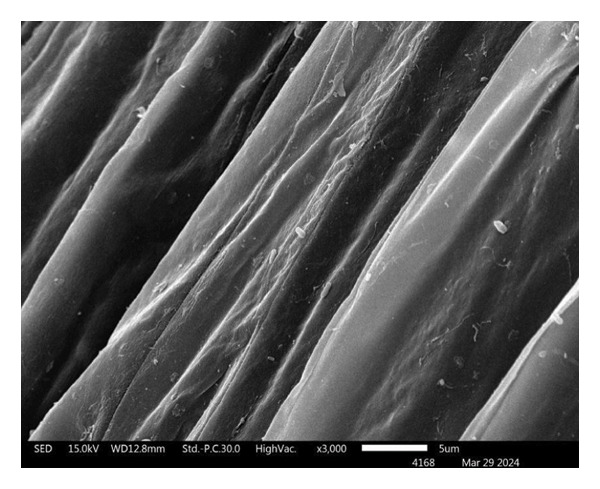
(a)

**Figure:**
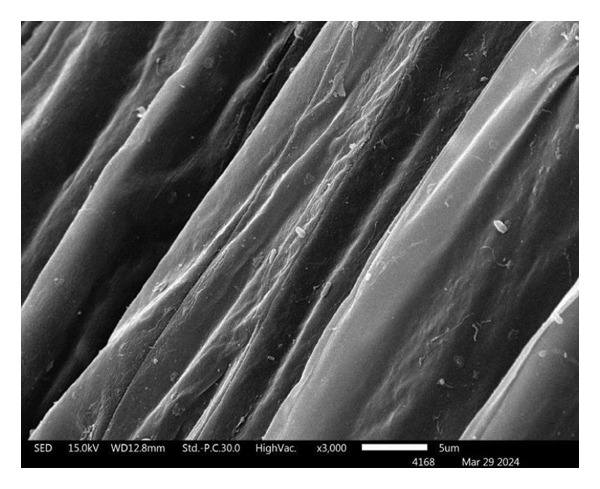
(b)

## 4. Discussion

The rise in global ambient temperatures has led to intense heat stress in agricultural crops, significantly affecting plant growth and development [7]. In uninoculated lettuce, morphological parameters reveal that high temperatures generally have a negative effect, as evidenced by reductions in fresh weight, dry weight, number of leaves, and root length (Figure 2). High temperature can damage the plant root resulting in reduced plant growth parameters including fresh weight, dry weight, and width [35]. The development of longer roots represents an important morphological adaptation that enhances plant resilience under stress conditions. Longer roots improve water and nutrient uptake efficiency, help maintain cellular turgor, and support overall physiological stability, thereby contributing to sustained plant growth and performance under adverse environmental conditions [36]. In this study, inoculated lettuce exhibited a 16% increase in root length compared to uninoculated controls. This enhancement is likely attributed to the beneficial effects of *S. thermocarboxydus* S3, which is known to produce IAA, a key phytohormone that promotes root development [29]. In addition, *S. thermocarboxydus* S3 was found to produce IAA at 45°C (data not shown). Recently, thermotolerant *Streptomyces* sp. AB‐11 was reported to tolerate temperatures up to 45°C and promote root development in chickpea [37]. Previous studies have confirmed that IAA improves root morphology, including enhancements in root length, root tip number, and root tip density [38, 39]. In addition, actinobacteria‐derived IAA has been shown to stimulate the formation of roots, which enables plants to absorb greater volumes of water and nutrients. In contrast, shoot length in uninoculated lettuce was longer than inoculated lettuce (Figure 2(b)). Abnormal shoot elongation is commonly observed under heat stress and is often considered as a stress‐induced response [15, 16, 40]. Interestingly, inoculated lettuce exhibited reduced shoot elongation compared to uninoculated controls under heat stress, suggesting that *S. thermocarboxydus* S3 may mitigate the adverse effects of elevated temperatures. This mitigation could be attributed to the bacterium’s ability to enhance physiological processes and regulate phytohormone balance, ultimately leading to improved plant performance and stress resilience [41].

The reduced levels of photosynthetic pigments observed in uninoculated lettuce (Figure 3(a)) are closely associated with diminished morphological growth, as evidenced by lower fresh and dry biomass and a reduced number of leaves (Figure 2). This decline suggests that elevated temperatures accelerate the degradation of chlorophyll and damage the photosynthetic apparatus, impairing the plant’s ability to capture and utilize light energy effectively [42]. In addition, the accumulation of ROS under heat stress contributes to pigment degradation by inducing oxidative damage. ROS such as singlet oxygen and H_2_O_2_ can disrupt chlorophyll molecules by attacking their porphyrin rings, ultimately leading to pigment loss and compromised photosynthetic efficiency [43–45]. Under optimal conditions, plants maintain a balance between the synthesis and degradation of photosynthetic pigments such as chlorophyll and carotenoids, ensuring pigment homeostasis. However, heat stress disrupts this balance by enhancing the activity of chlorophyll‐degrading enzymes, including chlorophyllase and chlorophyll peroxidase, thereby accelerating chlorophyll breakdown [44, 46, 47]. Similar reductions in photosynthetic pigments under high temperature stress have been reported in other crops, including sorghum [48], tomato [42], cabbage [49], cucumber [50], and paprika [51]. Interestingly, lettuce inoculated with *S. thermocarboxydus* S3 exhibited significantly higher chlorophyll and carotenoid levels compared to uninoculated controls (Figure 3(a)). This can indicate that *S. thermocarboxydus* S3 enhances photosynthetic efficiency and plant vitality via phytohormone production which can stimulate chloroplast development and delay chlorophyll degradation. Similar observations were found in a study using *Streptomyces* sp. to promote *Arabidopsis* sp. and *Brassica* sp. growth. An improved chlorophyll content was induced by protein accumulation including light‐harvesting chlorophyll (*Lhcb1*) protein [52]. The increase in carotenoid content may have contributed to the lower H_2_O_2_ content observed in the inoculated plants (Figure 3(c)) as carotenoids play a key role in antioxidant defense by quenching singlet oxygen and limiting ROS formation within chloroplasts [45, 53]. These findings suggest that *S. thermocarboxydus* S3 enhances heat stress tolerance in lettuce by maintaining photosynthetic pigment stability and mitigating oxidative damage, thereby supporting overall physiological balance under elevated temperatures. Similar effects have been reported in other plant species; for instance, inoculation with *Streptomyces* sp. significantly improved growth parameters, such as fresh weight, in *Arabidopsis thaliana* and *Brassica* species subjected to heat stress, indicating the potential of this genus to enhance thermotolerance through both growth promotion and stress mitigation mechanisms [52]. In addition to *Streptomyces*, *Bacillus* species have also been extensively studied for their ability to alleviate heat‐induced stress in plants and are widely utilized as bioinoculants. For example, *Bacillus cereus* SA1 has been shown to enhance soybean growth under heat stress by modulating phytohormonal levels and increasing the expression of heat shock proteins, thereby improving plant resilience [54]. Likewise, inoculation with *Bacillus* spp. has been reported to improve growth and root architecture in mustard under high‐temperature conditions, highlighting their role in promoting physiological adaptation and stress tolerance [55].

Plant growth‐promoting microorganisms (PGPMs) including actinobacteria can enhance plant tolerance to abiotic stresses through multiple physiological and biochemical mechanisms [56]. Under elevated temperatures, lettuce typically accumulates osmolytes such as proline and soluble sugars, which play crucial roles in osmotic adjustment, protein and membrane stabilization, and ROS detoxification. These compounds help maintain cellular homeostasis and support plant survival under heat stress conditions [57]. In the current study, inoculated lettuce exhibited significantly increased levels of proline and TSS (Figure 3(b)), indicating that these osmolytes contributed to improved stress tolerance. The correlation analysis revealed strong relationships among physiological and biochemical parameters in lettuce under heat stress. Notably, H_2_O_2_ levels were strong and negatively correlated with both proline and TSS. This suggests that as osmolyte levels increase, oxidative stress decreases, supporting the role of proline and sugars in ROS detoxification and osmotic adjustment. Interestingly, a strong positive correlation was found between proline and TSS, suggesting that these molecules may be coregulated in response to heat stress and act synergistically to protect cellular structures. Plant growth‐promoting actinobacteria have been reported to enhance proline accumulation in rice (*Oryza sativa*) [58, 59], wheat (*Triticum durum*) [60], and tomato [61] under abiotic stress condition. Similarly, inoculation with *Pseudomonas putida* AKMP7 in wheat under heat stress led to improved morphological growth and elevated levels of cellular metabolites, including proline and TSS [62].

PGPMs, including members of the phylum Actinomycetota, are increasingly recognized for their ability to enhance plant tolerance to abiotic stresses through diverse physiological and biochemical mechanisms [56]. Under elevated temperatures, plants activate adaptive responses, including the accumulation of compatible solutes such as proline and TSS, which are critical for osmotic adjustment, stabilization of proteins and membranes, and detoxification of ROS [57]. These compounds help maintain cellular homeostasis and mitigate the damaging effects of heat stress.

In the present study, *Streptomyces thermocarboxydus* S3‐inoculated lettuce plants exhibited significantly higher levels of proline and TSS compared to uninoculated controls (Figure 3(b)), suggesting that the inoculant enhanced osmoprotective capacity under thermal stress. This accumulation likely played a central role in protecting cellular structures and maintaining physiological processes. The observed increases align with previous studies demonstrating that PGPMs, particularly actinobacteria, promote proline accumulation in crops such as rice (*Oryza sativa*), wheat (*Triticum durum*), and tomato (*Solanum lycopersicum*) under abiotic stress conditions [58–61]. Similarly, inoculation with *Pseudomonas putida* AKMP7 in wheat enhanced both morphological parameters and osmolyte accumulation under heat stress [62], further supporting the role of microbial inoculants in inducing thermotolerance through metabolic modulation.

Correlation analysis (Figure 4) in the current study revealed a strong negative relationship between H_2_O_2_ levels and the concentrations of proline (*r* = −0.949) and TSS (*r* = −0.967), indicating that increased osmolyte production is associated with reduced oxidative stress. This suggests that osmolyte accumulation may contribute not only to osmotic balance but also to ROS detoxification. Furthermore, the significant positive correlation between proline and TSS (*r* = 0.992) implies a coordinated or synergistic regulatory mechanism, potentially driven by microbial priming, that enhances the overall stress resilience of the plant.

In addition to the role of osmolytes in heat stress mitigation, the production of nonenzymatic antioxidants also plays a crucial role in alleviating oxidative damage, primarily by neutralizing ROS such as H_2_O_2_. These antioxidants help prevent cellular damage to lipids, proteins, and nucleic acids, thus maintaining cellular integrity under stress conditions [45, 63, 64]. Lettuce is known to contain a diverse array of nonenzymatic antioxidants, including ascorbic acid, carotenoids, phenolic acids, and flavonoids, all of which contribute significantly to its oxidative stress defense system and overall plant resilience [65, 66]. In this study, the marked reduction in H_2_O_2_ content in *S. thermocarboxydus* S3‐inoculated lettuce suggests that oxidative stress was effectively mitigated. However, the relatively modest changes in DPPH and ABTS radical scavenging activity and total phenolic content indicate that nonenzymatic antioxidant production may not be the primary mode of ROS detoxification triggered by the inoculation of *S. thermocarboxydus* S3. Instead, the enhanced accumulation of osmolytes particularly proline and soluble sugars appears to play a more dominant role in protecting against oxidative damage as a strong negative correlation was observed between nonenzymatic antioxidants and osmolytes. These inverse correlations between osmolytes and lipophilic antioxidant activity may reflect distinct roles in mitigating heat‐induced oxidative stress. This suggests that increased osmolyte accumulation may support the plant’s antioxidant capacity indirectly, possibly by stabilizing cellular structures and reducing ROS generation. Conversely, the observed positive correlation between H_2_O_2_ and nonenzymatic antioxidant activity may reflect a stress‐induced response, where antioxidant mechanisms are upregulated in reaction to an increased oxidative pressure, rather than serving a preventive protective function. These findings highlight the complementary and potentially synergistic roles of osmolytes and nonenzymatic antioxidants in heat stress mitigation. The results also support the hypothesis that *S. thermocarboxydus* S3 contributes to lettuce thermotolerance by modulating key physiological pathways associated with osmotic balance and ROS detoxification.

The observed reduction in H_2_O_2_ content in *S. thermocarboxydus* S3‐inoculated lettuce is likely attributed to enzymatic antioxidant systems, particularly the activity of catalase. Catalase plays a crucial role in the detoxification of ROS by catalyzing the decomposition of H_2_O_2_ into water and oxygen, thereby preventing oxidative damage to cellular structures. Members of the genus Streptomyces, including *S. thermocarboxydus*, are well known for catalase production [67], and preliminary observations confirm that *S. thermocarboxydus* S3 is catalase positive. This suggests that the strain may enhance the plant’s oxidative stress tolerance primarily through catalase‐mediated ROS scavenging.

In addition to reducing oxidative damage, inoculation with *S. thermocarboxydus* S3 significantly improved lettuce growth and morphological traits under heat stress conditions without compromise in nutrient value (Table 1). This finding aligns with previous studies demonstrating that while biofertilizers and PGPMs enhance plant growth and yield, they may not significantly alter macronutrient or micronutrient accumulation. For instance, Demir et al. [68] reported similar results in lettuce and broccoli grown in greenhouse conditions with biofertilizers, where improved biomass did not correspond to significant changes in nutrient content. Similarly, the coinoculation of *Pseudomonas mendocina* and *Glomus intraradices* in lettuce under drought conditions improved proline and soluble sugar levels and enhanced antioxidant activity without significantly affecting nitrogen, P, K, or Ca concentrations [69]. These results suggest that the use of *S. thermocarboxydus* S3 as a bioinoculant in hydroponic systems effectively improves plant stress resilience and promotes growth performance under heat stress, without compromising nutritional quality. This is particularly important for leafy vegetables such as lettuce, where both yield and nutrient content are critical for marketability and consumer acceptance.

The successful colonization of *S. thermocarboxydus* S3 in the roots of hydroponically grown lettuce under heat stress was confirmed through both reisolation from the roots and SEM visualization (Figure 5). These findings strongly suggest that the observed improvement in lettuce growth under heat stress is, at least, influenced by root colonization and associated plant‐beneficial activities of *S. thermocarboxydus* S3. The seed coating method employed in this study effectively facilitated bacterial attachment and root colonization, consistent with earlier reports indicating that seed inoculation is a practical and efficient technique for delivering PGPBs [70]. Additionally, our previous investigation demonstrated that *S. thermocarboxydus* S3 can survive in hydroponic systems, where it successfully colonized roots despite a four‐log reduction in cell numbers [19]. This further validates the robustness of the strain under hydroponic and stress conditions. The ability of *S. thermocarboxydus* S3 to act as an endophyte is significant, as it allows bacteria to persist within plant tissues, providing a sustained source of growth‐promoting compounds and protection against abiotic and biotic stress. Previous studies have reported similar benefits from endophytic *Streptomyces* spp., including suppression of soilborne pathogens such as *Sclerotinia minor*, the causative agent of lettuce drop [71]. The colonization ability observed here confirms the classification of *S. thermocarboxydus* S3 functions as an endophytic PGPB, capable of enhancing plant performance under heat stress.

## 5. Conclusion

This study demonstrates that *S. thermocarboxydus* S3 significantly enhances the growth of hydroponically grown lettuce under heat stress conditions. This strain mitigates heat stress by promoting osmotic adjustment through the accumulation of compatible solutes such as proline and total soluble sugars, thereby safeguarding cellular structures against heat‐induced osmotic imbalance. Inoculation with *S. thermocarboxydus* S3 also reduced the accumulation of H_2_O_2_, a key reactive oxygen species, suggesting an enhancement of the plant’s antioxidant defense system and a reduction in oxidative stress. These findings are consistent with previous studies, which demonstrated the strain’s ability to support plant growth under various abiotic stress conditions, including high temperatures. Successful colonization of lettuce roots under elevated temperatures further confirms the strain’s thermotolerance and functional potential in hydroponic environments. Collectively, this study highlights *S. thermocarboxydus* S3 as a promising bioinoculant for improving heat resilience and overall productivity in hydroponic lettuce cultivation systems. Our findings also highlight the complex interplay among ROS accumulation, osmolyte synthesis, and antioxidant responses during heat stress adaptation in lettuce. Future research should aim to explore the specific enzymatic antioxidant pathways involved in stress alleviation, investigate the molecular signaling networks modulated by *S. thermocarboxydus* S3, and expand the application of this strain to other crops. These efforts will facilitate the development of robust, ready‐to‐use bioinoculant formulations tailored for hydroponic systems and other climate‐sensitive agricultural practices.

## Author Contributions

Conceptualization, Wasu Pathom‐aree; methodology, Benyapa Kitwetch, Yupa Chromkaew, and Wasu Pathom‐aree; investigation, Benyapa Kitwetch; data analysis, Benyapa Kitwetch, Yupa Chromkaew, and Wasu Pathom‐aree; visualization, Benyapa Kitwetch; writing–original draft, Benyapa Kitwetch; writing–review and editing, Yupa Chromkaew and Wasu Pathom‐aree; resource, Yupa Chromkaew and Wasu Pathom‐aree; supervision, Wasu Pathom‐aree; funding acquisition, Wasu Pathom‐aree.

## Funding

This research work was partially supported by Chiang Mai University, Thailand (grant number: RG05/2567).

## Disclosure

All authors have read and agreed to the published version of the manuscript.

## Conflicts of Interest

The authors declare no conflicts of interest.

## Data Availability

The data that support the findings of this study are available from the corresponding author upon reasonable request.
